# A simple dose of antibiotics: qualitative analysis of sepsis reporting in UK newspapers

**DOI:** 10.3399/bjgpopen20X101005

**Published:** 2020-01-22

**Authors:** Lynne Rush, Shona Hilton, Lisa McDaid

**Affiliations:** 1 Clinical Research Fellow, MRC/CSO Social and Public Health Sciences Unit, University of Glasgow, Glasgow, UK; 2 Professor of Public Health Policy, MRC/CSO Social and Public Health Sciences Unit, University of Glasgow, Glasgow, UK; 3 Professor of Social Sciences and Health, MRC/CSO Social and Public Health Sciences Unit, University of Glasgow, Glasgow, UK; 4 Professor of Social Sciences and Health, Institute for Social Science Research, University of Queensland, Brisbane, Australia

**Keywords:** sepsis, anti-bacterial agents, newspaper article, child, drug resistance, clinical decision-making

## Abstract

**Background:**

A recent drive to improve sepsis awareness has been accompanied by prolific media reporting about its management in children. Media reporting is known to influence public understanding of health issues and subsequent health-seeking behaviour.

**Aim:**

To examine UK newspaper representations of sepsis in children to better understand how the messages they convey may impact on parents' consulting behaviour and expectations about antimicrobial prescribing.

**Design & setting:**

Qualitative analysis of articles published in 12 UK newspapers from January 1988 to June 2018.

**Method:**

Thematic analysis of 140 articles about sepsis in children identified through a search on the Nexis database.

**Results:**

Reporting about sepsis in UK newspapers was characterised by emotive personal narratives about affected children who have suffered death or disability. These events were frequently presented as resulting from failings within the healthcare system that could have been avoided by early treatment. Health professionals were portrayed as inadequately prepared to recognise and manage sepsis, and as reluctant to prescribe antibiotics, even when necessary. Parents were positioned as advocates for their children, and as being ultimately responsible for ensuring that they receive appropriate treatment.

**Conclusion:**

This research identified messages about sepsis in the UK news media that could influence public attitudes about antibiotic prescribing in acute childhood illness. Public health communications about sepsis awareness must acknowledge the wider implications of unnecessary antibiotic use as a driver of antimicrobial resistance to reduce the risk of damaging efforts to promote rational prescribing.

## How this fits in

Efforts to reduce unnecessary prescribing of antimicrobials in primary care have previously focused on the minimal benefits associated with using antibiotics to treat minor childhood illness, such as upper respiratory tract infection. Recent news media reporting of sepsis in children challenges assumptions that non-treatment is unlikely to cause harm, documenting multiple occasions where children have died after reassurances from health professionals that their illness would resolve spontaneously. This represents a shift in messaging about antibiotic requirements that may impact on expectations about prescribing, with implications for antimicrobial stewardship strategies.

## Introduction

A recent drive to improve early recognition and management of sepsis has been accompanied by a recommendation from the National Institute for Health and Care Excellence (NICE) that encourages health professionals to consider sepsis in any patient presenting with symptoms of infection.^1^ The public have also been urged to have a low threshold for suspecting sepsis and to be prepared to challenge opinions of health professionals if necessary, centred around the campaign slogan *‘* *Just ask — could it be sepsis?* *’* in order to ensure that potentially lifesaving treatment is initiated, with a progressive increase in mortality reported for every hour that antibiotics are delayed.^2–4^ While increased awareness is essential to improve outcomes, there are practical considerations about how this guidance may be enacted in a primary care setting, where most antibiotics are prescribed.^5^ Avoidance of unnecessary antibiotic use is central to strategies to reduce spread of antibiotic resistant infections, which have been predicted to cause 10 million deaths annually by 2050.^6,7^

Initiatives to promote better awareness of sepsis have been accompanied by prolific media reporting about individuals who have died or experienced serious disability, often as a result of misdiagnosis. Although the vast majority of sepsis cases occur in adults, a previous quantitative analysis study by the present authors demonstrated that 46% of articles published in 12 UK newspapers between January 1988 and June 2018 that contained references to individuals who had been affected were about infants or children.^8^ Particularly prominent was the case of 1-year-old William Mead, who died of sepsis associated with an untreated streptococcal infection. An enquiry by NHS England concluded that pressure on GPs to avoid prescribing antibiotics may have contributed to his death.^9^ *The Daily Mail* subsequently launched a campaign entitled *‘* *End the Sepsis Scandal* *’*, supported by Mead’s mother and The Sepsis Trust. Following Mead’s death, a plethora of articles appeared in this paper and others about children who had died in similar circumstances.

Personal narrative stories involving children are of interest for several reasons. First, presentation of health issues as posing a particular risk to young children has been identified as a ‘fright factor’ that increases audience concern.^10^ Second, clinical factors associated with assessing children in primary care make them an important population where avoidable prescribing may occur; respiratory tract infections are the most frequently encountered presentation in primary care among children under 5 years of age and the most common indication for antibiotics.^3^ Decisions about antibiotic prescribing are often complex and influenced by non-clinical factors, including clinician perception of parental expectations.^4^ Furthermore, NICE guidance on sepsis acknowledges that assessing young children is potentially difficult due to age-related differences in physiological parameters that form an important part of clinical assessment, interpretation of which may be more challenging for GPs without specialist paediatric experience.^1^

Media reporting of health issues has been demonstrated to impact on health behaviour, for example, in relation to uptake of immunisation and screening programmes.^11,12^ Furthermore, audiences may overestimate the risk of rare events in response to the volume of media coverage received.^13^ Media reporting about sepsis in children thus has the potential to impact on primary care attendance and expectations about antibiotic prescribing.

The aims of this article were: to use a thematic analysis of a subset of articles about sepsis from the present authors’ previous quantitative analysis^8^ containing narratives about affected children to identify messages presented by UK newspapers about recognition and management of sepsis in children; and to hypothesise about what this might mean for parental health-seeking behaviour and attitudes to antibiotic use.

## Method

**Figure 1.**
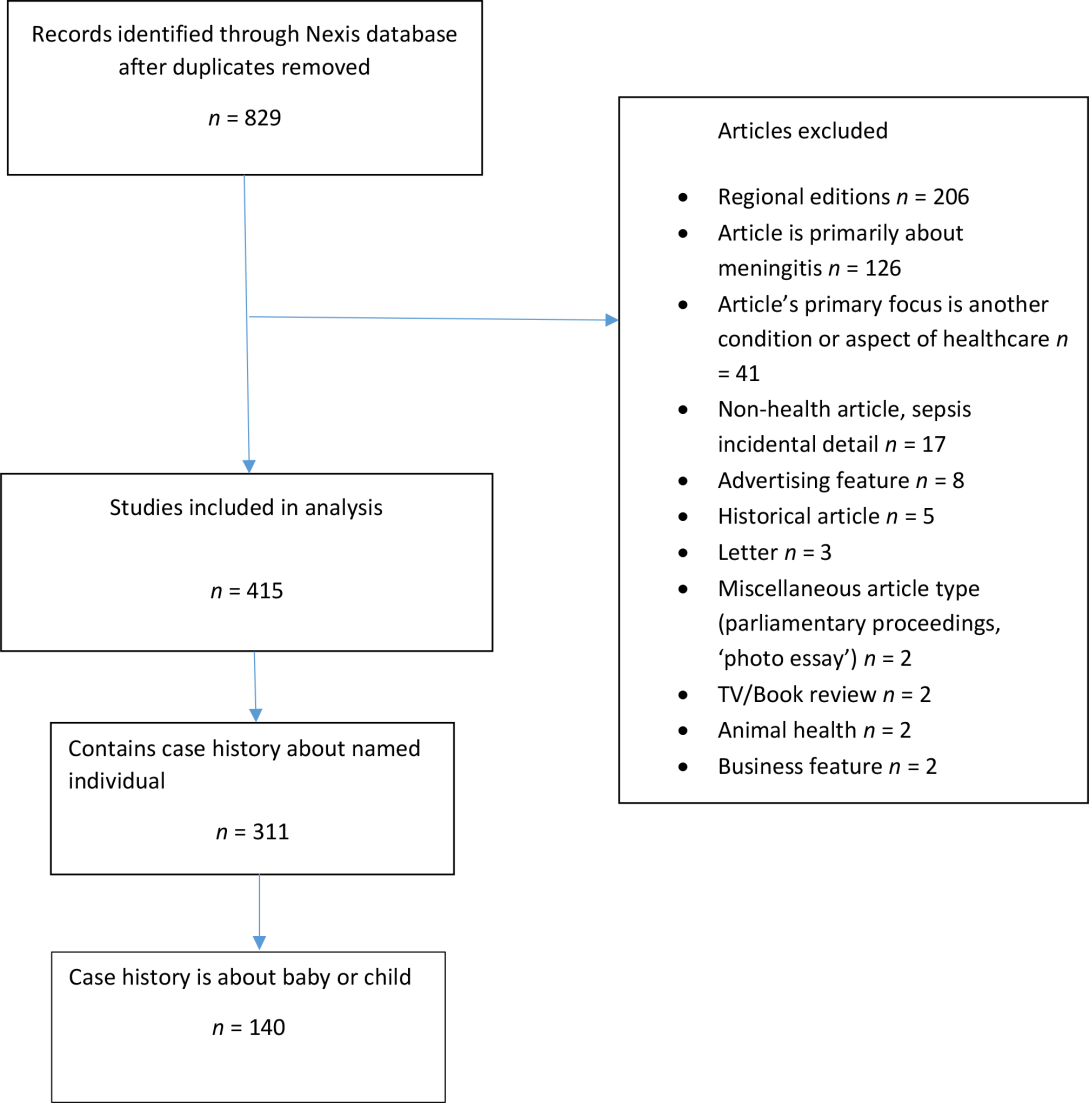
PRISMA diagram of study inclusion

The search process used to identify articles about sepsis included in the present authors’ previous quantitative analysis has been described elsewhere^8^ and is summarised in Figure 1; the authors searched the Nexis database, applying no date restrictions, for articles containing three or more mentions of ‘sepsis’, ‘septicaemia’, or ‘blood poisoning’ within 12 national UK newspapers reflecting diverse readership in terms of age, social class, and political alignment.^8,14^ One-third (34%) of the articles in this sample identified children (defined as aged 0–16 years) who had been affected by sepsis, and these were imported into the qualitative analysis software package (NVivo version 12.0).

To code the content of these 140 articles, the authors used a thematic analysis approach, as described by Braun and Clarke.^15^ One author generated initial codes, informed by the findings of the quantitative analysis and by novel themes identified from close reading of the articles. Two further authors independently coded a random subsample of 10 articles. The suitability of the draft coding frame, including duplications or omissions, was discussed by all three reviewers and was subsequently revised and refined, with the final version consisting of five overarching themes: sepsis; victims; the healthcare system; families; and antibiotics (see Supplementary Box 1). All articles were then re-coded using the revised coding frame to ensure consistency. Analysis of the coded content is presented alongside typical quotations.

## Results

In the 140 articles analysed, 52 different babies or children affected by sepsis were named, 38 of whom died. Some cases, in particular the deaths of Samuel Morrish and William Mead, were particularly prominent and were used repeatedly to illustrate health service failings, even in articles that were primarily about other children. The distribution of articles across newspaper titles is shown in Table 1. The earliest article about sepsis in children identified on the Nexis database was published in February 1995.

**Table 1.** Summary of articles in sample

**Title**	**Articles, *n* (%)**
**Broadsheet**	
Guardian or Observer	11 (8)
Telegraph or Sunday Telegraph	8 (6)
**Middle market**	
Daily Mail or Mail on Sunday	97 (70)
Express or Sunday Express	6 (4)
**Tabloid**	
Mirror or Sunday Mirror	9 (6)
The Sun or News of the World	9 (6)

### Sepsis: the silent killer

Sepsis was presented as a condition that is silent, fast, and common. Frequent references to sepsis as *‘* *the silent killer* *’* conveyed the often apparently trivial nature of initial symptoms including *‘* *simple sniffles* *’*, *‘* *common colds* *’*, and *‘* *tummy upsets* *’* that in retrospect were harbingers of the terrible events to follow.

Warnings about the speed with which familiar childhood illnesses can render the sufferer moribund seemed intended to evoke dread; sepsis *‘* *can develop rapidly following even the mildest of infections* *’* (*Daily Mail*, 9 Feb 2016), taking hold with *‘* *devastating swiftness* *’* (*Daily Mail*, 14 June 2016) and *‘* *terrifying speed* *’* (*Daily Mail*, 16 Feb 2018), leading to *‘* *rapid death* *’* (*Express*, 9 Feb 2016) in children who had appeared no more than mildly unwell just hours before. One parent, mother of 3-year-old Morrish, described how it took the life of her child in *‘* *what felt like an instant* *’* (*Daily Mail*, 14 Sep 2016).

Along with Mead’s story, reporting of the enquiry into Morrish’s death appeared to signal a paradigm shift in how sepsis was represented. According to his parents, his death in 2010 was initially presented to them as unavoidable: *‘We were told that Sam had died of something rare, fast-acting, hard to spot and therefore very hard to treat*’ (*Guardian*, 19 July 2016); however, a subsequent investigation by the Healthcare Ombudsman in 2014 concluded that his death would have been easily prevented with antibiotics and criticised the initial internal enquiry for failing to be open to this conclusion.^16^ Prior to this case, sepsis was framed in the media as a tragic but uncommon occurrence, worthy of attention precisely because of its nature as an exceptional event, as illustrated by the following quotes from parents who had lost children:

*‘The doctors said this could happen in one in a million cases and unfortunately we were that one.*’ (*Daily Mail*, 23 May 2006)

*‘People need to know it's extremely serious but also extremely rare.*’ (*Express*, 1 Jan 2010)

Following the enquiry into Morrish’s death, sepsis was no longer framed as rare and unfortunate but as *‘* *the leading cause of avoidable death in the UK* *’* (*Daily Mail*, 23 May 2017), claiming *‘* *35 000* *lives a year, including* *1000* *under-fives* *’* (*The Sun*, January 27 2016).

### Victims: needless deaths of little ones

Although many articles acknowledged that sepsis is indiscriminate and can affect anyone, children were identified as a group at increased risk:

*‘It can strike previously healthy patients of all ages, but is most common in young children, pregnant women, the elderly and those with underlying illness.* *’* (*Daily Mail*, 3 March 2016)

Framing of children’s deaths from sepsis as largely avoidable was a recurrent theme, declared via alarming and emotive headlines such as, ‘*Sepsis and the little ones who should never have died’* (*Daily Mail*, 15 March 2016).

This focus on avoidable deaths was reinforced by stakeholders within the health sector, notably The Sepsis Trust, whose opinions featured frequently. Bold statements were made about the potential for awareness campaigns to improve outcomes, with a substantial proportion of recent deaths seemingly directly attributed to perceived delays by the UK Government:

*’In all, says The UK Sepsis Trust,* *55 000* *adults and children have died of sepsis (in the past* *14* *months* *) since Mr Hunt pledged to act, of whom at least* *16 000* *could still be alive today if loved ones had known what to look for.*’ (*Daily Mail*, 15 March 2016)

### Healthcare system: shameful blunders

Descriptions of the health service’s management of sepsis were almost universally critical. Morrish’s death was described as the result of a *‘* *catalogue of errors* *’* (*Telegraph*, 18 Dec 2017), while the multiple contacts that Mead had with health services preceding his death was referred to as ‘*a series of shameful blunders* *’* (*Daily Mail*, 26 Jan 2016). Numerous articles detailed similar instances of children sent home from primary care or Accident and Emergency (A&E) departments with misplaced reassurance. Frequently, these failings were attributed to inadequate knowledge and understanding:

*‘… awareness* [of sepsis] *among medical professionals and the public is worryingly poor, delaying life-saving treatment.*’ (*Daily Mail*, 3 May 2016)

Other articles blamed attitudinal factors; *‘* *patronising* *’* doctors were described as giving *‘* *cursory examinations* *’* (*Daily Mail*, 18 Aug 2017) and concerned parents dismissed as *‘* *overprotective* *’* (*Daily Mail*, 27 Jan 2016) or *‘* *paranoid* *’* (*Daily Mail*, 7 March 2018). For another child who underwent multiple amputations after developing sepsis as a complication of minor burns, staff were *‘* *too arrogant* *’* to consider sepsis as a diagnosis, even after his mother raised it as a possibility (*Mail on Sunday*, 21 Aug 2016).

When adverse outcomes occurred, there were no assurances in the articles that clinical governance would prevail. Criticism of the NHS’s procedure for investigating fatalities ranged from accusations of apathy to blatant attempts to deceive. The most widely reported failing by an identifiable healthcare professional concerned Bawa Garba, convicted of manslaughter following the death of 6-year-old Jack Adcock. Despite subsequent recognition that complex systemic factors contributed to Adcock’s death, the General Medical Council’s description of Bawa Garba’s care as *‘* *truly, exceptionally bad* *’* was widely reported.^17^

Of the 140 articles, just six (4%) contained praise for the health service’s management of sepsis in children. However, even those articles with positive outcomes aimed to caution parents; one mother, who according to the article now works with The Sepsis Trust to raise awareness, warned: ‘*It could have turned out so differently. It could have been someone who wasn't confident enough to point out it could be sepsis.’* (*Guardian*, 22 Jan 2016).

### Parents: it’s not too late for you

The devastation that sepsis has wrought on the lives of affected families was apparent within the highly emotive accounts contained in many articles:

*‘Our life now is empty, painful and will never be the same again. To say that we miss him does not do justice to our strength of feeling* *— Jack was an amazing son and one in a million.’* (*Daily Mail*, 5 Nov 2015)

*‘I feel like my heart has stopped beating. When I wake up, I think I can’t go through a whole day. I’m devastated. I just feel empty.’* (*Express*, 25 Jan 2011)

*‘The hardest thing was closing the* [coffin] *lid and saying my last goodbye. When you lose a child, you lose their future and your future. There’s no tomorrow. You just get through to the end of each day.’* (*Daily Mail*, 27 Oct 2016)

Alongside powerful descriptions of grief were insights into the regret that parents experienced when faced with the possibility that different actions could have altered outcomes. For Mead’s mother, *‘the worst thing in the world is knowing that his life could have been saved.*’ (*Daily Mail*, 26 Jan 2016).

In every article in the sample, parents reported that they had consulted for medical advice, sometimes repeatedly, but the message from those who have lost children to sepsis was that this may be insufficient. This was reinforced by journalistic framing, as illustrated in the following editorial excerpt:

*’… it doesn't matter how diligent or loving you are, you still can't protect your child from institutionalised incompetence* […] *the death of William Mead should never be forgotten. Not just because it exposes gaping holes in NHS care; because it unites us all as parents.*’ (*Daily Mail*, 27 Jan 2016)

In this way, responsibility shifts to parents, obligating them as advocates for their children, a role sanctioned by both experts and by fellow parents. Ron Daniels, CEO of The Sepsis Trust and a key actor in the drive to improve sepsis awareness, wishes to ‘empower’ parents to recognise symptoms so that they can seek treatment immediately and raise the question of sepsis if doctors do not: *‘* *We would urge parents to always trust their instincts and ask a medical professional,* *"* *Could it be sepsis?"'* (*Guardian*, 15 Dec 2011). The mother of a baby who died after repeated reassurance that there was nothing seriously wrong warned: *‘* *A mother's intuition is key — the doctor is not always right* *’* (*Daily Mail*, 18 Aug 2017).

Several articles described the guilt endured by parents who survive their children. The UK actor Jason Watkins has spoken extensively about the loss of his 2-year-old daughter, Maude Watkins, and urged fellow parents to have a low threshold for action: *‘My message to anyone is, even if there are just one or two symptoms, get straight down to A* *&* *E*‘ (*Express*, 9 Feb 2016). His wife, Clara Francis, was described as unable to shake the conviction that she was somehow to blame: *‘You have one job, to stop your child dying, and I couldn’t do that.*’ (*Daily Mail*, 9 Feb 2018).

The need to prevent others falling victim to the same fate imbues warnings to other parents with an imploring quality and sense of urgency, captured in this plea from Mead’s mother: *’I will never hear my sweet child say,* *“Mummy, I love you.*“ *I will never know the man that William would have grown to be. So please, it is too late for me to* *“think sepsis*“ *but it's not too late for you.*’ (*Guardian*, 15 Dec 2016).

Parental instinct was therefore presented as key to safeguarding children from this fast-moving, terrifying threat. Yet this concept was poorly defined, and even parents who have experienced loss did not necessarily feel better equipped to recognise when to act. Mead's mother is described as *'beset by worries'* when her younger son is unwell: '*Until* *Arthur can talk I'll always assume the worst if he grizzles or has a cough or a temperature. Even though I know so much about sepsis now, there's always the fear I'll miss something.* *'* (*Daily Mail*, 27 Oct 2016).

### Antibiotics: the simple solution

The relative ease with which terrible outcomes could have been prevented by *‘* *a simple dose of antibiotics* *’* is alluded to in several articles, and is alluring in its apparent simplicity. The consequences of treatment delays were recounted repeatedly, giving a sense of pivotal moments where treatment decisions meant the difference between life and death:

*‘A simple jab of antibiotics would have saved him.’* (*Mail on Sunday*, 29 Nov 2015)

*‘Knowing that a simple course of antibiotics could have saved him is something his parents will have to live with for the rest of their lives.’* (*Daily Mail*, 27 Oct 2016)

*‘Blessing Matia’s death could have been prevented with a simple dose of antibiotics if medical staff had been able to identify the condition.’* (*Daily Mail*, 3 Dec 2016)

*‘Experts said he would “probably” have survived if he had been given a simple dose of antibiotics.’* (*Telegraph*, 13 Feb 2016)

One article described how Mead’s *‘* *fate was sealed* *’* by failure to refer him to hospital where he could have received antibiotics that may have saved him (*Guardian*, 26 Jan 2016). For Maude Watkins, *‘* *the window of opportunity to save her life was fast closing* *’* as her condition deteriorated following discharge from hospital the previous day (*Daily Mail*, 9 Feb 2016). On reattending, she was given antibiotics but *‘it was almost certainly too late for* [the antibiotics] *to have had any impact on the condition.* *‘* The suggestion here is that treatment at an earlier stage may have averted her death; however, the absence of a clear indication for antibiotics in the form of confirmed bacterial infection presents a conflict for doctors who were described as under ‘*constant pressure* *’* not to prescribe (*Guardian*, 26 Jan 2016). One article described the conflicting priorities that doctors working in primary care face:

*‘* [GPs] *are in an impossible bind. On one hand* […] *they are heavily criticised for not prescribing antibiotics in time. While on the other they are constantly criticised by health officials like the Chief Medical Officer Dame Sally Davies — dubbed the nanny-in-chief for her diktats on how we should lead our lives — for prescribing antibiotics* *.’* (*Daily Mail*, 27 Jan 2016)

This same article, while giving the semblance of a balanced representation of the dilemma facing GPs, concluded that, given the findings of the enquiry into Mead’s death, *‘surely it is better to err on the side of caution?* *’*

Notably, no articles that referred to pressure on GPs to reduce antibiotic prescribing connected this with efforts to reduce antibiotic resistance. Instead, it was positioned alongside issues about managing resources, so that withholding antibiotics was placed on a par with avoiding referring patients to crowded emergency departments. This frames antibiotics as a treatment that patients may be denied despite the benefits they might confer rather than because of the harm that unnecessary treatment may cause. The six articles (4%) that did mention growing resistance to antibiotics did so in relation to its potential contribution to the overall burden of sepsis, failing to acknowledge the paradoxical messages sent out about prescribing:

*’Numbers* [of sepsis cases] *have leapt more than* *50* *per cent* *in five years, with experts partly blaming GPs* *'* *over-prescription of antibiotics*.’ (*Daily Mail*, 3 March 2016)

This creates a potentially confusing message, with health professionals to blame as individuals for failing to prevent deaths from sepsis by prescribing antibiotic treatment where needed, but also, as a collective, driving resistance through unnecessary prescribing.

## Discussion

### Summary

The qualitative analysis of the reporting of childhood sepsis in UK newspapers revealed a picture presented to a public audience of a vast and previously unseen death toll, the result of a failing health service that cannot be relied on to provide necessary care. Previously healthy children were presented as disproportionately at risk, which is out of keeping with the known epidemiology of sepsis.^18^ Parents were positioned as ultimately responsible for ensuring that health professionals recognise when their child’s symptoms may be early indicators of sepsis, ensuring access to early antibiotics that may otherwise be withheld.

### Strengths and limitations

To the authors’ knowledge, this study is the first to examine UK newspapers’ reporting about childhood sepsis. Analysis benefited from a broad search period which allowed the authors to explore representations of sepsis spanning a 25-year period, identifying a shift in framing from 2010.

Twelve news titles were searched; however, the authors acknowledge that their findings include a preponderance of illustrative quotes from *T* *he* *Daily Mail*, which published a high volume of stories about sepsis in children as part of its *‘* *End the Sepsis Scandal* *’* campaign. Although this newspaper dominated reporting that used personal narratives, the content of messaging about sepsis was consistent across genres and news brands, as demonstrated in the authors’ previous quantitative analysis.^8^ Although the authors did not include online versions of news articles, the content of these has been demonstrated to be broadly similar to print versions.^19^

### Comparison with existing literature

Reducing unnecessary antimicrobial prescribing is vital to combat the spread of drug resistant infections, but no studies were identified that demonstrate whether antimicrobial prescribing for children in primary care can be safely reduced without impacting on the incidence of sepsis. Existing literature has focused on the incidence of specific complications following upper respiratory tract infections but did not include sepsis as an outcome measure.^20^ A recent study investigating the impact of withholding antibiotics in older patients with urinary tract infection identified a significant association with sepsis and all-cause mortality.^21^ It is well recognised that prescribing in primary care is influenced by a host of non-clinical factors; sustained reporting about deaths in previously healthy children that may have been avoided by *‘* *a simple dose of antibiotics* *’* adds another facet to the challenge of upholding antimicrobial stewardship in primary care.^22–25^

Findings from the present study demonstrate a shift over a relatively short period of time in how deaths from sepsis are represented in news reports, from something tragic but unpredictable to the result of dire and inexcusable failures in diagnosis and management. Other commentators have described how changing societal perceptions of risk and a shift in focus from the ‘probable’ to the ‘possible’ have extended to the parental role, which now includes being conscious of potential harm from all sources and taking all possible measures to avoid these.^26–28^ The positioning in the media of children as a group who are particularly vulnerable to harm has been identified as a powerful way of mobilising public emotion and an important driver in gaining political support for action on other public health issues, for example, legislation on smoking in cars.^29–31^

This shift in risk communication does not reflect true changes in risk, but rather an expansion in the range of diseases captured under the banner of 'sepsis', one that serves to inflate the apparent risk to individuals. Throughout history, certain diseases have demonstrated a capacity to develop meanings that extend beyond the disease itself. Susan Sontag has described how some diseases, for example cancer and AIDS, have functioned as metaphors for what is morally or socially wrong:

*‘Nothing is more punitive than to give a disease meaning — that meaning being invariably a moralistic one. Any important disease whose causality is murky and for which treatment is ineffectual tends to be awash in significance* […] *the disease itself becomes a metaphor. Then, in the name of the disease, that horror is imposed on other things.* *’* ^32^

For sepsis, the potential causes are so myriad as to include any childhood illness, and the underlying causative organisms rarely alluded to. In effect, this is the reverse process that Sontag describes as having occurred for understandings of other conditions, notably cancer, where progressive understandings of malignancy at a molecular level have resulted in its recognition as not one disease but many, simultaneously lessening its capacity to create stigma.^32^ In hospital settings, improved capture of sepsis through more consistent allocation of diagnostic codes is essential to improve monitoring and surveillance.^33,34^ However, for a public audience, attempts to ‘rebrand’ sepsis as one disease overlooks the natural history of the underlying infections, creating fear and uncertainty, and may be less effective in furthering understandings of childhood infectious disease than for conditions where the disease process is better defined, such as meningococcal disease.

### Implications for practice

It is likely that increased awareness of sepsis will reduce associated mortality through earlier diagnosis and management; however, the potential for unintended consequences from press coverage that is almost unwaveringly negative in tone must be considered. The almost universal criticism of medics and allied health professionals in managing sepsis seems incongruous with constructions of sepsis as difficult to diagnose (even for experienced clinicians) and as frequently requiring something additional to assessment of the facts presented (for example, parental intuition). The importance placed on intuition, so difficult to define and yet advocated as a vital tool by experts and fellow parents, conflicts with professional understandings of sepsis as a condition whose diagnosis hinges on specific and measurable physiological parameters.

Ron Daniels speaks of the thousands of lives that could have been saved if an awareness campaign had been in place earlier. Though highly effective in putting sepsis on the heath agenda, this message may be viewed as reductionist, oversimplifying the ease with which early symptoms of sepsis can be accurately identified. This has the potential to place unsustainable demands on services by parents concerned that any minor illness may be indicative of sepsis, as well as creating unnecessary anxiety for parents and practitioners. Here, ‘awareness’ seems to be presented as comparable with, for example, a novel cancer drug or introduction of a mass vaccination programme. Modelling mortality outcomes on predicted impacts of greater awareness fails to acknowledge the heterogeneity of sepsis as a condition, or the complexity of decision-making when confronted with symptoms that *might* be early indicators. Furthermore, parents who have lost children to sepsis are, by their own admission, ill-positioned to make confident decisions when confronted with subsequent episodes of childhood illness. It seems, then, that the solution *‘* *Just ask — could it be sepsis?* *’* may be overly presumptive in its simplification of a complex condition.

Concerns have been raised about an observed increase in defensive practice following the high-profile reporting of Bawa Garba’s conviction for manslaughter.^35^ It is unclear whether defensive practice will translate into defensive prescribing, but the potential for this to impact on strategies to reduce antimicrobial resistance must be considered as a possible unintended consequence of sepsis awareness. Positioning of health professionals as ill-prepared to recognise and manage sepsis, and of parents as obligate advocates for their children, may increase the likelihood of antimicrobial prescribing that is not clinically justified. Failure by UK journalists to acknowledge wider societal considerations surrounding antimicrobial prescribing and the importance of preserving the effectiveness of antibiotics is concerning. Ultimately, positioning of sepsis as a condition that disproportionately affects healthy children has the potential to damage efforts to promote the antimicrobial stewardship that is an essential part of the strategy to reduce antimicrobial resistance.^7,36^ This study suggests that there is scope to incorporate messages about the risks associated with overuse of antibiotics into the media conversation about sepsis to further public understandings about the need for a rational approach to prescribing decisions.
